# Are Liver Pericytes Just Precursors of Myofibroblasts in Hepatic Diseases? Insights from the Crosstalk between Perivascular and Inflammatory Cells in Liver Injury and Repair

**DOI:** 10.3390/cells9010188

**Published:** 2020-01-11

**Authors:** Lindolfo da Silva Meirelles, Renan Fava Marson, Maria Inês Gonzalez Solari, Nance Beyer Nardi

**Affiliations:** 1 PPGBioSaúde and School of Medicine, Lutheran University of Brazil, Av. Farroupilha 8001, 92425-900 Canoas, RS, Brazil; 2 PPGBioSaúde, Lutheran University of Brazil, Av. Farroupilha 8001, 92425-900 Canoas, RS, Brazil; 3 Institute of Cardiology of Rio Grande do Sul, Av Princesa Isabel 370, 90620-001 Porto Alegre, RS, Brazil

**Keywords:** perivascular cells, pericytes, hepatic stellate cells, inflammation, macrophages, Kupffer cells, liver injury, liver diseases

## Abstract

Cirrhosis, a late form of liver disease, is characterized by extensive scarring due to exacerbated secretion of extracellular matrix proteins by myofibroblasts that develop during this process. These myofibroblasts arise mainly from hepatic stellate cells (HSCs), liver-specific pericytes that become activated at the onset of liver injury. Consequently, HSCs tend to be viewed mainly as myofibroblast precursors in a fibrotic process driven by inflammation. Here, the molecular interactions between liver pericytes and inflammatory cells such as macrophages and neutrophils at the first moments after injury and during the healing process are brought into focus. Data on HSCs and pericytes from other tissues indicate that these cells are able to sense pathogen- and damage-associated molecular patterns and have an important proinflammatory role in the initial stages of liver injury. On the other hand, further data suggest that as the healing process evolves, activated HSCs play a role in skewing the initial proinflammatory (M1) macrophage polarization by contributing to the emergence of alternatively activated, pro-regenerative (M2-like) macrophages. Finally, data suggesting that some HSCs activated during liver injury could behave as hepatic progenitor or stem cells will be discussed.

## 1. Introduction

It is estimated that approximately two million deaths per year worldwide are due to liver diseases, including cirrhosis, viral hepatitis, and liver cancer [1]. Although accurate incidence, prevalence, and mortality data are not available for a large number of countries, recent reviews have shown that vaccination, screening, and antiviral treatment campaigns have reduced the disease burden in some regions of the world [2,3]. However, increased use of injection drugs and alcohol, changes in diet and life habits, as well as prevalence of obesity and diabetes, tend to further increase the global burden of acute and chronic liver disease.

Acute liver failure is a rare, life-threatening condition following severe hepatic injury. It can be caused by a variety of events leading to damage of liver cells, including viral infection and toxic drug effects. Hepatic ischemia, drug-induced liver injury, and viral and autoimmune hepatitis are among the most prevalent causes [4]. Most of these are resolved by eliminating the stimuli, which results in recovery of normal function and morphology. Rates of survival have improved in recent years through improved intensive care and liver transplantation procedures.

In chronic liver diseases, which include viral infections, nonalcoholic steatohepatitis, alcoholic liver disease, and autoimmune diseases, necrosis and inflammation may progress to liver fibrosis and cirrhosis [5]. The inflammatory response to liver injury is well studied, and basic principles have been established. The initial stimulus activates liver-resident macrophages or Kupffer cells (KCs), which induce the expression of adhesion molecules such as CD44 by the endothelium [6] and recruitment of circulating blood cells. Infiltrating neutrophils, through molecular mechanisms not yet fully understood, clear pathogens and debris and undergo apoptosis [7]. Circulating monocytes are also massively recruited, which mature into macrophages or dendritic cells to repair the injured tissue [8]. The molecular pathways responsible for these events, including a great number of cytokines, chemokines, and receptors, have been extensively investigated [9] and may provide potential therapeutic targets for liver diseases.

The course of chronic liver diseases depends mostly on the resolution of inflammatory processes. Prolonged inflammation results in extensive deposition of extracellular matrix (ECM) consisting of proteoglycans, glycoproteins, fibrous proteins, and collagen-α1 [8,10]. Reversal of the inflammatory process returns the liver to its healthy state. Different cell types are involved in the processes of homeostasis, progression, and regression of liver diseases. Understanding their biology and function, as well as their interactions, is critically important for preventing and treating chronic liver diseases.

## 2. Macrophages and Liver Injury

Macrophages, myeloid immune cells present throughout the body, are key regulators of liver fibrosis deposition and resolution [11]. As phagocytic cells, they represent one of the most ancient elements in the evolutionary process of innate immunity. Unicellular organisms have receptors capable of recognizing foreign elements for phagocytosis. Since the earliest evolutionary stage, multicellular animals such as sponges, worms and cnidarians, have had phagocytes similar to macrophages which have repair and/or fight functions [12]. Mechanisms include the recognition of cellular debris, foreign material, or pathogens, which are then ingested and degraded.

Hepatic macrophages, key elements in maintaining homeostasis as well as in inflammation and repair of the liver, are a heterogeneous population consisting of cells derived from different origins. In homeostasis, KCs are predominant, but during injury circulating monocytes are recruited and differentiate into macrophages. A third, less known population of peritoneal macrophages may be recruited to the liver in specific types of injury. Although no specific markers are available to discriminate between resident and monocyte-derived macrophages, human KCs can be identified by expression of CD14, CD16, and CD68 [13]. The antigens CD163L and CLEC5A have been proposed to discriminate between the two populations in humans [14]. Murine KCs are positive for CD45, CD68, F4/80, and C-type lectin domain family 4, member f (Clec4F), and express intermediate levels of CD11b [15,16]. Numerous reviews are available on the origin, biology, and function of these cell populations during homeostasis and disease [17,18,19].

A recent study identified new populations of nonparenchymal cells in healthy and cirrhotic human livers, by analyzing the individual transcriptomes of more than 100,000 cells [20]. Ten clusters of mononuclear phagocytes, seven clusters of endothelial cells, and four clusters of mesenchymal cells were identified. Among these clusters, a scar-associated TREM2^+^CD9^+^ subpopulation of macrophages and ACKR1^+^ and PLVAP^+^ subpopulations of endothelial cells were expanded in cirrhotic livers. Profibrogenic pathways, including TNFRSF12A, PDGFR, and NOTCH signaling were observed in the fibrotic niche with multilineage modeling of interactions between fibrotic macrophages, endothelial cells, and mesenchymal cells.

KCs, which account for nearly 30% of the non-parenchymal cells in the liver and around 85% of the tissue macrophages in the body, self-renew from liver-resident cells originated from the fetal yolk sack [21]. They are non-migratory cells, occupying a fixed position in the hepatic sinusoidal endothelium. In the healthy liver, KCs exert important roles as a primary line of defense against intestine-derived pathogens and by maintaining tolerance to foodborne and bacterial antigens from the intestine [22,23]. Although KCs are able to process and present antigens [24], they express MHC -II and costimulatory molecules in significantly lower levels than dendritic cells [25]. Furthermore, they secrete high levels of the T-cell inhibitory molecule PDL-1 and IL-10, which induces expansion and activation of regulatory T cells [26].

During liver injury, the inflammatory pathway in KCs is activated through toll-like receptor (TLR) pattern recognition receptors. These cells rapidly produce cytokines and chemokines such as IL-1b, tumor necrosis factor TNF-α, CCL2, and CCL5, resulting in the recruitment of circulating monocytes [27,28]. Two different populations of blood monocytes have been identified in mice according to the expression level of Ly6C (Gr-1). Ly6C^hi^ monocytes also express CCR2, rapidly infiltrate the tissue, and are primarily responsible for acute inflammation, while LY6C^low^ cells express CX3CR1 and may serve as precursors for dendritic cells [29,30]. In humans, the subpopulations of circulating monocytes are defined as classical (CD14^++^ CD16^−^), intermediate (CD14^+^ CD16^+^), and non-classical (CD14^dim^ CD16^+^) populations [31].

Macrophage activation states have been traditionally defined as classical (M1) or alternative (M2) activation [32]. Considering this terminology, M1 cells are activated by LPS, IFN-γ, or high-mobility group protein 1, are microbicidal and tumoricidal, and favor Th1 pro-inflammatory responses. M2 macrophages favor Th2 responses, have anti-inflammatory effects and promote tissue repair. They can be further subdivided into M2a, M2b, and M2c, which are activated by IL-4/IL-13, LPS/IL-1β, and IL-10/glucocorticoids, respectively [33]. M1 and M2 cells release numerous inflammatory or anti-inflammatory cytokines and chemokines [17]. Ly6c expression levels have also been used to characterize liver macrophages, with a correspondence between the activities of Ly6C^hi^ and Ly6C^low^ cells and M1 and M2 macrophages, respectively [34].

More recently, the definition of M1 and M2 cells has been questioned [8,19,35]. Macrophages are plastic populations and can display various functions simultaneously or in sequence. The possibility to reprogram or repolarize macrophages into different phenotypes and functions [36] holds great potential for targeting these cells for the treatment of liver diseases [11].

When liver cells interact normally between themselves and with their microenvironment, homeostasis is maintained and injuries are repaired, with completion of the three phases normally seen in inflammatory processes. As mentioned above, in the early proinflammatory step, KCs recognize the injury and contribute to the recruitment of blood monocytes, which then mature into macrophages. As the inflammatory response proceeds and the injury is resolved, macrophages switch to a reparative phenotype and, in the final stage, normal tissue architecture is restored. Failure in any of these steps may result in fibrosis, with excessive deposition of collagen and other extracellular matrix proteins. A better understanding of these mechanisms, and other cell types that may affect the process, is critically important for the prevention and management of liver diseases. In the next section, the roles of perivascular cells during the evolution of liver injury will be discussed, with emphasis on their interactions with inflammatory cells.

## 3. Perivascular Cells in Liver Injury

Cirrhosis, a late stage of various forms of chronic liver disease, is characterized by vascular dysfunction and extensive fibrosis, which compromises molecule exchange between the blood and liver parenchymal cells. Common outcomes include parenchymal extinction and portal hypertension [5]. Activation of hepatic stellate cells (HSCs) is involved with liver fibrosis, which also includes other cell types able to produce collagen such as portal fibroblasts and bone marrow-derived myofibroblasts [37]. This HSC characteristic can be replicated in vitro, as HSCs become proliferative, express alpha-smooth muscle actin (αSMA), and secrete abundant amounts of collagen when placed in culture [38]. Since fibrosis is an important aspect of various liver conditions including cirrhosis, mechanisms underlying HSC activation have been the subject of studies aiming to find molecular targets to inhibit liver fibrosis [39].

HSCs are considered to be a liver-specific pericyte [40,41] and, like pericytes in other tissues, are physically connected with endothelial cells and directly interact with them in a reciprocal manner [42]. Unlike pericytes in most other tissues, however, HSCs are not embedded in a thick basement membrane; instead, the abluminal side of HSCs is included in a plasma-filled compartment between the sinusoids and the liver parenchyma, the space of Disse [43]. It is important to notice that HSCs are not the only type of pericyte in the liver. Liver pericytes positive for CD146, but negative for the classical HSC markers αSMA and glial fibrillary acidic protein (GFAP), have been described too [44], and pericytes without specific HSC characteristics exist in the portal vessels and central vein [45]. Likewise, perivascular cells in the liver do not comprise only pericytes, as other mesenchymal cells including fibroblasts and smooth muscle cells can be found around larger blood vessels [46]. KCs, in turn, are associated with endothelial cells in the lumen of sinusoids to play their role as a liver-specific type of macrophage [47].

KCs have receptors for pathogen-associated molecular patterns (PAMPs) and damage-associated molecular patterns (DAMPs) [48]. Lipopolysaccharide (LPS) is an example of Gram-negative bacteria-derived PAMP that triggers KC activation by activation of toll-like receptor (TLR) 4 [49]. DAMPs, which comprise a number of molecules released by necrotic cells, can cause KC activation in various types of sterile liver injury through activation of TLR4 as well [50]. KCs activated by LPS [51] or DAMPs released by liver cells in response to acetaminophen-induced toxicity [52] secrete interleukin- (IL-) 18, a cytokine that induces interferon-gamma production by T cells [53] and, consequently, directs a type I immune response. Additional inflammatory mediators secreted by KCs stimulated through TLR4 include IL-8 and macrophage chemoattractant protein-1 [MCP-1, also known as C chemokine ligand (CCL2)] [54], and CXC chemokine ligand 2 (CXCL2) [55], all of which are involved with the recruitment of neutrophils [56]. In addition, PAMPs, DAMPs, and inflammatory cytokines lead to activation of endothelial cells [57], which expose adhesion molecules that lead to leukocyte docking and consequent infiltration into the tissue [58]. Infiltrating neutrophils phagocytose microorganisms such as bacteria, if present, and secrete microbicidal proteins and reactive oxygen species (ROS) that can cause bystander damage [59,60]. These infiltrating neutrophils also produce interleukin-17A, which has been shown to promote HSC activation [61]. Influenced by the inflammatory milieu, infiltrating monocytes differentiate into proinflammatory macrophages that produce transforming growth factor-beta (TGF-β), which, together with other stimuli such as signaling through platelet-derived growth factor receptor β (PDGFRβ) [62], contributes to HSC activation [27], proliferation, and consequent deposition of extracellular matrix in the space of Disse.

Excessive deposition of extracellular matrix in the space of Disse is detrimental to liver function because it hinders the exchange of molecules between the blood and the parenchyma, which impairs liver function and favors portal hypertension. In view of this, strategies to block HSC activation could represent a way to prevent or minimize loss of hepatic function after liver injury. Consequently, pharmacological inhibition of HSC activation has been attempted using molecules that inhibit signaling through tyrosine kinase receptors (as is the case for PDGFRβ) [63] or TGF-β receptors [64] in in vitro and preclinical models of liver injury. Unfortunately, translation of the beneficial results found in these models into the clinic is difficult because tyrosine kinase and TGF-β receptors are expressed by many other cell types throughout the body, which means that many side effects are expected when these receptors are pharmacologically inhibited.

### 3.1. Pericytes Favor Inflammation at the Early Moments after Injury

One of the consequences of the knowledge on the events involved in liver injury depicted above is the perception that the main role of HSCs in these processes is to become extracellular matrix producers. However, further information on HSCs and pericytes from other tissues suggest the roles of these cells during tissue injury and repair are far greater than that. For example, HSCs express TLR4 and secrete IL-8 and MCP-1 when stimulated by LPS [54] and other PAMPS [65]. Likewise, pericytes from the lungs of rats also secrete inflammatory cytokines when stimulated by PAMPs [66]. Increased production of proinflammatory molecules by pericytes in the lungs of mice can be detected as early as six hours after the experimental administration of LPS [67]. Another reported characteristic of pericytes in other tissues is the ability to respond not only to PAMPs but also to DAMPs by secreting proinflammatory cytokines, as demonstrated in pericytes from the lungs of mice [67] and humans [68], and in human placental pericytes [69]. Secretion of inflammatory cytokines by HSCs after stimulation with DAMPs has not been demonstrated in vivo yet, even though an HSC cell line has been shown to exhibit greater expression of activation markers after stimulation with the DAMP high-mobility group box 1 (HMGB1) [70]. In in vitro migration assays, human placental pericytes stimulated with the DAMP N-formyl-methionyl-leucyl-phenylalanine (fMLP) have been found to attract neutrophils by secreting macrophage inflammatory factor (MIF) and IL-8 (CXCL8), and monocytes by secreting MIF and CCL2 [69]. Accordingly, an intradermal injection of fMLP promotes increased extravasation of neutrophils and their retention around pericytes in postcapillary venules as assessed using intravital microscopy in the skin of mice genetically labeled to allow observation of pericytes and neutrophils [69]. It is important to highlight that the signaling molecules mentioned above are just a few of a number of proinflammatory mediators known to be produced by pericytes. For example, pericytes also produce CXCL1 and IL-6 [69]. Additional information on other proinflammatory molecules secreted by pericytes can be found elsewhere [71]. Further evidence indicates that, besides physically interacting with myeloid leukocytes by means of adhesion molecules such as intercellular adhesion molecule 1 (ICAM-1), pericytes increase neutrophil survival and stimulate the activation of both neutrophils and monocytes [69]. The abovementioned information indicates that, at the onset of tissue injury, DAMP-stimulated pericytes behave as proinflammatory cells by stimulating infiltration, survival, and activation of inflammatory cells. This behavior is further highlighted by the demonstration that selective depletion of HSCs improves the outcome in experimental models of liver injury in mice [72,73]. Consequently, further research on the proinflammatory properties of liver pericytes, particularly HSCs, is warranted in order to find molecular targets for intervention in acute liver injuries.

As seen above, HSCs may actively participate in the recruitment of inflammatory cells in liver injury, a behavior further demonstrated for pericytes in other tissues. The influx of inflammatory cells triggers other important mechanisms required for the resolution of liver injury, including limiting fibrosis. In experimental liver injury induced by D-galactosamine, the number of HSCs, identified as cells positive for the low-affinity p75 nerve growth factor (NGF) receptor (p75NTR, also known as LNGFR or CD271), is increased 36 h after the injury [74]. Interestingly, increased numbers of p75NTR^+^ pericytes have been observed after cardiac injury in humans and mice too [75]. In mice, p75NTR has been shown to be essential for HSC differentiation into myofibroblasts [76], even though absence of functional p75NTR does not preclude HSC activation [77]. Quiescent human HSCs express low levels of p75NTR; on the other hand, the myofibroblast-like cells derived from them express higher levels of this molecule [77]. During liver diseases, hepatocytes produce NGF [78,79] as well as its precursor form, pro-NGF [80]; binding of NGF to p75NTR induces apoptosis in HSCs [81]. Cultured liver myofibroblasts also produce pro-NGF [77]. After liver injury, the hepatic levels of pro-NGF are highest at the peak of liver fibrosis and fall during the subsequent recovery period [77]. This decrease in pro-NGF is connected to the action of macrophages that are located at fibrotic areas; these scar-associated macrophages (SAMs) secrete matrix-metalloproteinase 7 (MMP7), which is responsible for conversion of pro-NGF to NGF by means of cleavage [77]. Consequently, SAMs play an important role in limiting fibrosis by favoring NGF-dependent apoptosis in myofibroblasts. Interestingly, selective depletion of SAMs during the recovery phase after liver injury leads to impaired reduction of fibrotic tissue [82].

### 3.2. Hypothesis: Activated HSCs Resemble Mesenchymal Stromal Cells, and Contribute to Macrophage Polarization toward an M2-like Phenotype

Various types of evidence suggest that pericytes behave as mesenchymal stem cells in different tissues [83], including the liver [84]. Pericytes, including HSCs, have been shown to give rise to cultured mesenchymal stromal cells (MSCs) [85,86,87], which are able to differentiate into bone, cartilage, and fat cells in vitro and secrete a number of paracrine molecules that have trophic and immunomodulatory properties [88]. Consequently, MSCs seem to represent a cultured form of activated pericytes. Indeed, when the global gene expression profile of cultured pericytes and MSCs obtained from human adipose tissue were compared, they were found to be almost identical [87]. Therefore, pericytes are postulated to become activated after tissue injury and, in this activated, proliferative state, secrete paracrine molecules that reduce inflammation and apoptosis [89,90]. The similarities between cultured pericytes and MSCs extend to mesenchymal differentiation ability [84,87,91] and suppression of activated T cells, as demonstrated for cultured pericytes from the retina [92], adipose tissue [87], and liver (HSCs) [91]. Another important similarity between MSCs and cultured HSCs is their ability to stimulate monocytes to develop into alternatively activated macrophages characterized by the production of high amounts of TGF-β in vitro [93,94]. In considering all these resemblances, it is possible that activated HSCs represent not only precursors of myofibroblasts, but also highly plastic cells similar to MSCs that can provide trophic support to parenchymal cells and even differentiate into liver-specific cells under particular circumstances, as documented [85]. Since the interactions between macrophages and HSCs are crucial for their activation and the downstream events that may result in restoration of liver function or cirrhosis, further investigation on the molecular interactions between these two cell types may hold the key to liver regeneration in liver diseases.

In the first moments after an injury event, the molecular crosstalk between perivascular cells and macrophages in liver diseases results in HSC activation. In this regard, amphiregulin secreted by KCs after experimentally induced liver injury in mice has been shown to promote HSC activation by signaling through the epidermal growth factor receptor [95]. More recently, tissue-resident macrophages were shown to trigger the activation of pericytes after lung injury by secretion of amphiregulin [96]. In that work, amphiregulin was found to activate cell surface integrin-α_V_ complexes which, in turn, convert latent TGF-β into its active form with consequent promotion of proliferation of pericytes and their differentiation into myofibroblast-like cells [96]. In the absence of hepatocyte growth factor derived from endothelial cells, perivascular fibroblasts upregulate expression of NADPH oxidase 4 [97], which has been shown to generate ROS in monocytes and macrophages [98] and can increase damage by ROS production. During the progression of experimental liver injury, TGF-β is observable in myofibroblasts and macrophages in fibrotic areas, as well as in hepatocytes; in contrast, TGF-β is no longer observable in these regions during the recovery phase [82]. A fact that may go unnoticed is that TGF-β suppresses type 1 inflammation [99] and is secreted by alternatively activated (pro-regenerative, M2-like) macrophages that develop during wound healing as opposed to classically activated (proinflammatory) macrophages activated by PAMPs and DAMPs present at the early stages of this process [100] (and which produce amphiregulin [101]). Additionally, TGF-β has been shown to contribute to alternative activation in macrophages [102,103]. In other words, the overall macrophage phenotype at the injured liver changes as the injury progresses and wound healing takes place, and perivascular cells or their progeny are involved in this change. In line with this, a hepatoprotective effect of fibrosis has been linked to alternatively activated macrophages (M2-like macrophages) that develop in the fibrotic liver and protect hepatocytes from apoptosis [104]. The ability of activated HSCs to shift macrophage polarization toward an M2-like phenotype, however, is detrimental in cases of hepatic cancer, as the immunosuppressive actions of these macrophages hinder the elimination of cancer cells by the immune system which contributes to poor prognosis [105].

**Figure 1:**
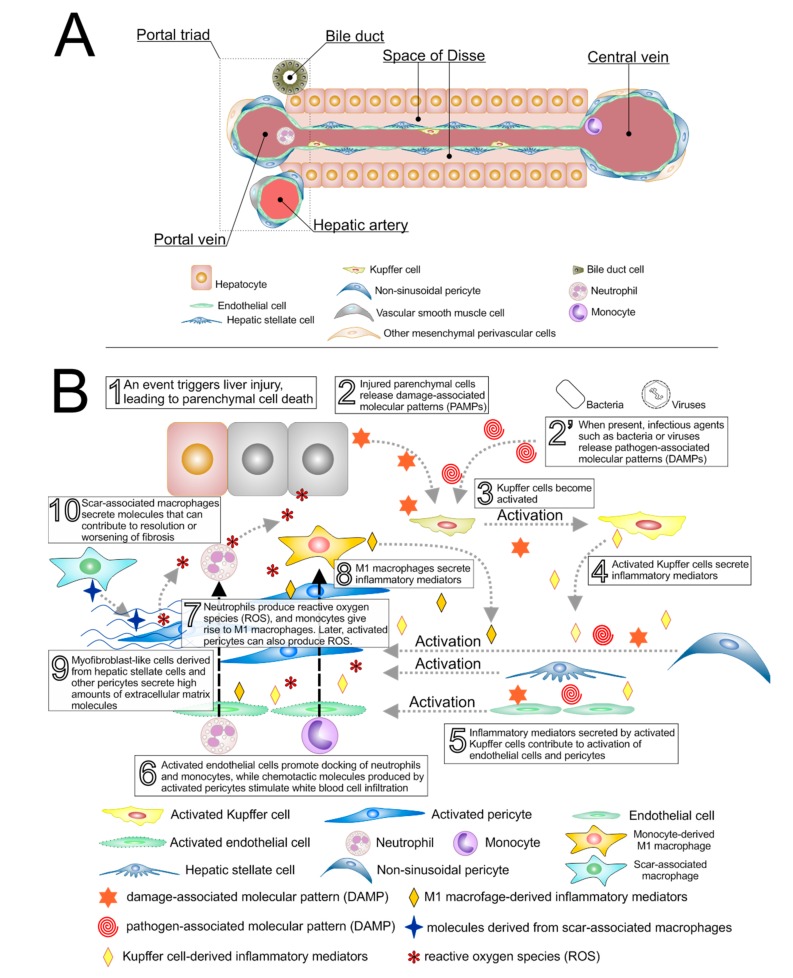
Interactions between inflammatory cells, perivascular cells, and endothelial cells at the onset and after a liver injury event. (**A**) Depiction of cells and structures in the liver under normal circumstances; (**B**) Sequence of events involved in the progression of various types of liver injury. Hepatocyte injury (1) causes release of damage-associated molecular patterns, DAMPs (2). These DAMPs lead to Kupffer cell activation (3) and consequent release of inflammatory mediators (4). Kupffer cells can also be activated by pathogen-associated molecular patterns (PAMPs) derived from pathogenic bacteria or viruses (2′). Inflammatory mediators secreted by activated Kupffer cells trigger activation of endothelial cells and perivascular cells (hepatic stellate cells and, to a lesser extent, non-sinusoidal pericytes) (5). DAMPs and PAMPs also contribute to the activation of endothelial cells and pericytes. Activated endothelial cells promote docking of circulating neutrophils and monocytes, which are stimulated to infiltrate into the tissue owing to the chemo-attractive inflammatory mediators present there (6). Neutrophils secrete reactive oxygen species, ROS (7), while infiltrating monocytes differentiate into proinflammatory (M1) macrophages, which secrete additional inflammatory mediators (8). In the presence of inflammatory mediators, hepatic stellate cells lose their morphological characteristics and, within a few days from the initial injury event, become proliferative cells that produce large amounts of collagen-rich extracellular matrix (ECM), which accumulate in the space of Disse (9) and hinder molecule exchange between the blood and the parenchyma. These cells, also known as myofibroblasts, also contribute to the generation of ROS, which causes further damage in hepatocytes and endothelial cells, and TGF-β, which contributes to modulation of the macrophage phenotype (see text for details). Finally, (10) macrophages that develop along this process (scar-associated macrophages) may take up phenotypes that secrete molecules that help revert fibrosis or may favor perpetuation of fibrosis. During this process, some activated pericytes may give rise to parenchymal cells (see text for details).

**Figure 2:**
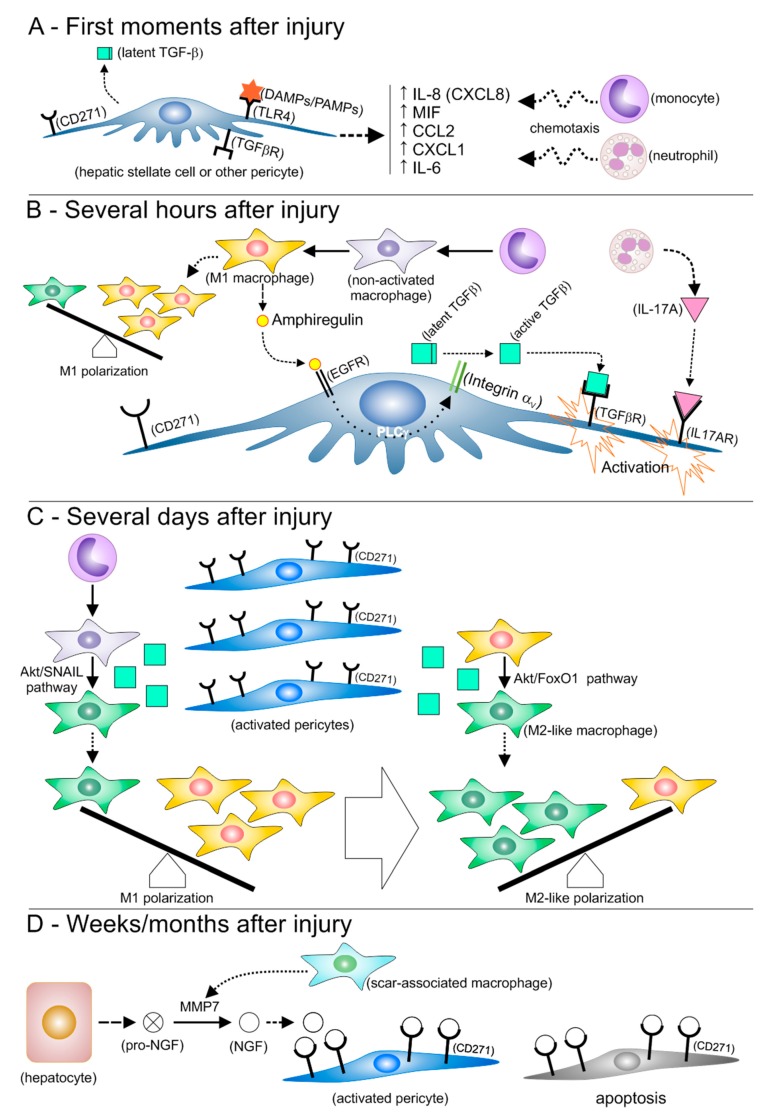
Molecular events mediating the interactions between inflammatory cells and perivascular cells soon after a liver injury event and thereafter. (**A**) Binding of DAMPs or PAMPs to toll-like receptor 4 (TLR4) increases secretion of a number of molecules that are chemotactic to neutrophils and monocytes, which favors their extravasation. (**B**) M1 macrophages develop from infiltrating monocytes, contributing to an M1 polarization. Neutrophils secrete interleukin 17A (IL-17A), while amphiregulin secreted by M1 macrophages leads to conversion of the latent form of TGF-β into its active form. Binding of IL-17A and TGF-β to their receptors triggers the activation of hepatic stellate cells and other pericytes. (**C**) Several days after the initial injury event, the number of activated pericytes increases, with consequent production of increased levels of active TGF-β. TGF-β promotes the acquisition of an M2-like phenotype by macrophages that have not acquired an effector phenotype, as well as by M1 macrophages. Macrophage polarization then shifts from M1 to M2-like. (**D**) During the weeks and months that follow, scar-associated macrophages develop and produce matrix metalloproteinase 7 (MMP7), which converts pro-nerve growth factor (pro-NGF) produced by hepatocytes into NGF. NGF binds to the low-affinity NGF receptor CD271, which is abundant on the surface of myofibroblast-like cells derived from activated pericytes and makes a number of these cells undergo apoptosis.

Based on the above, the crosstalk between perivascular cells and macrophages during liver injury and repair can be envisioned as the events that follow, which are depicted in Figure 1 and further detailed in Figure 2. Firstly, PAMPs and/or DAMPs lead to endothelial and activation and instigate perivascular cells to secrete inflammatory chemoattractants, with consequent infiltration of inflammatory cells and production of ROS. Classically activated macrophages, whether resident or derived from monocytes, promote activation and proliferation of perivascular cells, which will give rise to myofibroblast-like cells that secrete abundant amounts of extracellular matrix proteins. These myofibroblast-like cells produce the active form of TGF-β, which, together with neutrophil-derived ROS [106], contributes to macrophage polarization toward a TGF-β-producing, pro-regenerative phenotype (Figure 2). The main putative pathway involved with an M2-like polarization is Akt [102], including Akt/SNAIL [103] and Akt/FoxO1 [107]. Myofibroblasts expressing high levels of p75NTR undergo apoptosis triggered by NGF, which is produced by cleavage of parenchymal cell-derived pro-NGF operated by macrophage-derived MMP7. Not all myofibroblast are expected to undergo apoptosis in the short-term, and continuous liver damage fuels this whole sequence, increasing fibrosis. Under this perspective, not all activated liver pericytes will become myofibroblasts, and there is some evidence that some of these non-myofibroblastic activated HSCs may behave as stem cells during wound healing in the liver [108].

## 4. Conclusions

Even though it is established that perivascular cells, mainly HSCs, are precursors of myofibroblasts responsible for fibrotic lesions in various types of liver disease, other roles played by liver pericytes seem to be underappreciated. Pericytes in the liver and other tissues play an active role in the development of inflammation in the first hours after tissue injury by attracting and facilitating the infiltration of inflammatory cells into the tissue. However, further data suggest that, as the healing process evolves, the progeny of HSCs take up a yet poorly recognized role of modifying the initial proinflammatory macrophage polarization toward pro-regenerative phenotypes. Additionally, results from in vivo experiments suggest that some activated HSCs may behave as stem or progenitor cells after liver injury, as proposed for pericytes in other tissues. Consequently, regarding HSCs as just myofibroblast precursors in liver diseases does not seem to be appropriate. In fact, further basic research on the interactions between HSCs and inflammatory cells may provide new information on molecular targets for intervention in hepatic diseases.
